# Factors associated with low levels of lumbar strength in adolescents in Southern Brazil☆

**DOI:** 10.1016/j.rpped.2014.05.001

**Published:** 2014-12

**Authors:** Diego Augusto Santos Silva, Eliane Cristina de Andrade Gonçalves, Leoberto Ricardo Grigollo, Edio Luiz Petroski

**Affiliations:** a Universidade Federal de Santa Catarina (UFSC), Florianópolis, SC, Brazil; b Fundação Educacional Unificada do Oeste de Santa Catarina (UNOESC), Chapecó, SC, Brazil

**Keywords:** Motor activity, Physical fitness, Low back pain, Adolescent health, Spine, Pain

## Abstract

**OBJECTIVE:**

To determine the prevalence and factors associated with low levels of lumbar strength in adolescents.

**METHOD:**

This was a cross-sectional study involving 601 adolescents, aged 14 to 17 years, enrolled in public schools in the western region of Santa Catarina State - Southern Brazil. Lumbar strength was analyzed by the lumbar extension test developed by the Canadian Society of Exercise Physiology, which proposes different cutoffs for boys and girls. Independent variables were sex, age, socioeconomic status, dietary habits, alcohol consumption, physical activity, and aerobic fitness. For data analysis, univariate and multivariate logistic regression were used, with significance level of 5%.

**RESULTS:**

The prevalence of low levels of lumbar strength was 27.3%. The population subgroups most likely to present low levels of lumbar strength were females (OR: 1.54, 95% CI : 1.06 to 2.23), adolescents with low levels of aerobic fitness (OR: 2.10, 95% CI: 1.41 to 3.11) and the overweight (OR: 2.28, 95% CI: 1.35 to 3.81).

**CONCLUSION:**

Almost one-third of the studied students have low levels of lumbar strength. Interventions in the school population should be taken with special attention to female adolescents, those with low levels of aerobic fitness, and those with overweight, as these population subgroups were most likely to demostrate low levels of lumbar strength.

## Introduction

Lower back pain, also known as "lumbago," is one of the most common musculoskeletal discomforts in adolescents and adults. However, the diagnosis is not always specific, and it is considered a multifactorial disease.^1^

Studies have shown a great variability of the estimated prevalence of lumbar complaints among young individuals, ranging from 1.1%^2^ to 66.2%.^3^ In Brazil, the studies have shown that the prevalence of adolescents with back pain ranged from 19.5%^4^ to 31.6%.^5^

The main causes of back pain in students may be related to increasing weight bearing, such as carrying heavy school backpacks inadequately, as well as stay in the sitting position for prolonged periods, high-intensity sports practice, and low lumbar strength. The burden of school backpacks, physical inactivity, excessive sports practice, and low levels of lumbar strength, when associated with obesity and inadequate postural habits, maximize the occurrence of low back pain.^6^^,^ 7

A cohort study carried out by Vital, et al^8^ evaluated 215 Portuguese students and concluded that low levels of muscular strength of the trunk extensors/flexors are associated with greater prevalence of low back pain and low flexibility of the hamstrings. Similar results were found in longitudinal studies, such as that by Lee, et al,^9^ in Japan, with 67 young individuals between 17 and 19 years, and in that by Sjølie & Ljunggren,^10^ in Norway, with 85 adolescents with a mean age of 14 years, who observed association of low levels of lumbar strength with high BMI and a family history of lower back pain. In Brazil, a cross-sectional study by Martins, et al,^11^ with 60 adolescents aged 15-18 years, demonstrated that those with lumbar hyperlordosis had low levels of lumbar strength.

The disorders caused by low levels of lumbar strength ultimately affect the quality of life of adolescents and other health-related aspects, and result in considerable public expense for the treatment and rehabilitation of injuries.^7^^,^ 9 However, there have been no studies in Latin America, with a representative sample of adolescents, which simultaneously analyzed the association of low levels of lumbar strength with demographic variables, socioeconomic factors, and lifestyle. Thus, considering that low levels of lumbar strength are associated with the onset of lower back pain and that early onset of disorders in the spinal column affects some of these individuals in adulthood, it is important to analyze factors associated with low levels of lumbar strength to prevent the emergence of risk factors for lower back pain. Thus, the aim of this study was to determine the prevalence and factors associated with low levels of lumbar strength in adolescents.

## Methods

The study was conducted in Western Santa Catarina, which consists of 13 municipalities. The western region is one of mesoregions of the Brazilian state of Santa Catarina and has a human development index (HDI) of 0.807.^12^ The main town is Joaçaba, considered the economic and political center of Midwestern Santa Catarina, with an estimated 25,322 inhabitants.^13^

This was a cross-sectional study of a school-based population, targeted to schoolchildren aged 14 to 17 enrolled in public schools, and was conducted in the second semester of 2008. The study was approved by the Ethics Committee on Human Research of Universidade do Oeste de Santa Catarina (Opinion number 079/08).

All 13 towns of the region were included in the sampling plan, which was performed in two stages: 1) stratified by public high schools (n=18), and 2) conglomerate of classes. In stage 1, only schools with more than 150 enrolled students were considered (n=17). Moreover, in the towns that had more of one school, the one with a higher number of students was chosen. Thus, the largest school in each municipality was analyzed. In stage 2, all high school students who were present in class on the day of data collection were invited to participate in the study. 

For the sample size calculation, this study adopted an unknown prevalence for the outcome (equal to 50%), tolerable error of 5%, confidence level of 95%, and a design effect of 1.5, adding 10% for possible losses and refusals. Considering that in Midwestern Santa Catarina 4,582 schoolchildren constituted the high school population, a sample of 585 adolescents was estimated. However, as all adolescents belonging to the conglomerate of classes were invited to participate in the study, the final sample consisted of 635 adolescents.

When considering the parameters of study power of 80%, confidence level of 95%, and the number of subjects in each category of independent variables, the sample size could detect an odds ratio >1.4 and <0.6 as risk and protective factors, respectively, in the crude analysis. 

Adolescents were defined as eligible if they were enrolled in public state schools, were present in the classroom on the day of data collection, and if they were 14-17 years of age. If the adolescent did not want to participate, it was considered a refusal. Sample loss was considered if one or more of the variables analyzed in this study were not completed in the questionnaire or in the case of non-performance of one or more physical tests.

The dependent variable was lumbar strength. The test used to assess levels of strength in the lower back is an isometric test proposed by the Canadian Society of Exercise Physiology through the set of tests described in the Canadian Physical Activity, Fitness and Lifestyle Approach (CPAFLA).^14^

The isometric trunk extension-flexion test was carried out on a bench on which the assessed individual lies down in the prone position, resting only the legs and hips on the bench, with legs supported by padded belts around in the posterior regions of the thigh and leg. At the sign of the examiner, the individual raises the trunk to a horizontal plane with the legs, staying for as long as possible in this position. The test was terminated when the individual let the trunk go back to the original position or to a maximum of three minutes (180 seconds). Male and female adolescents were classified as having adequate or inadequate (low) levels of lumbar strength according to the cutoff points suggested in the CPAFLA, which vary according to gender and age.^14^

Socio-demographic data(gender, age, and income) were collected through a self-administered questionnaire in the classroom. The age variable was collected continuously and subsequently categorized as 14-15 years and 16-17 years. The economic level was identified through the Brazilian Association of Research Companies questionnaire,^15^ which divides the Brazilian population into five economic classes, in order of decreasing purchasing power (A1, A2, B1, B2, C1, C2, D, and E). In the present study, categories A1, A2 and B1 were grouped and considered as high economic level; B2, as mid-level; and C1, C2, and D as low level. No student in the class was rated as E. This classification was chosen due to sample homogeneity in these three categories. 

The students' dietary habits were recorded based on an item that integrates into the Fantastic Lifestyle Questionnaire, translated into Brazilian Portuguese and validated in Brazil.^16^ The item had the following sentence: "I eat a balanced diet." This item had the following response options: 1) almost never; 2) rarely; 3) sometimes; 4) with relative frequency; and 5) almost always. A balanced diet was considered for those who answered options 4 and 5. The tool explains what a balanced diet is, with different servings of grains, cereals, fruit, vegetables, dairy, meat, and similar items, which vary according to age and gender.

The behavior in relation to physical activity was analyzed by the Stages of Behavior Change (SBC) tool related to physical activity,^17^ which classifies individuals into one of five stages: (1) Pre-Contemplation (the individual does not want to modify his behavior in the near future); (2) Contemplation (there is intention to change, but not immediately); (3) Preparation (individuals who are not engaged in regular physical activity, but are willing to engage in the next 30 days); (4) Action (regularly active for less than six months); and (5) Maintenance (active on a regular basis for at least six months). 

Based on the SBC response, the students were classified in a behavior state of "insufficiently active", stages 1, 2 and 3, and "active", stages 4 and 5. Evidence of efficacy, sensitivity, and specificity of this classification can be obtained in the literature.^18^ Physical activity was considered as any bodily movement produced by skeletal muscles and resulting in energy expenditure above resting levels.^19^ Regular physical activity was considered as the recommendations for adolescents that state that everyone should participate in activities of at least moderate intensity, for at least 60 minutes at least five days a week.^20^

Excessive alcohol consumption, characterized by "binge drinking", which represents excessive drinking by young individuals, was investigated by one item of the Fantastic Lifestyle Questionnaire.^16^ The item is as follows: "I drink more than four alcoholic drinks on a single occasion." This item had as response options: 1) Almost daily; 2) With relative frequency; 3) Occasionally; 4) Almost never; and 5) Never. Individuals who responded options 4 and 5 were considered as non-heavy drinkers, whereas the others were considered heavy drinkers.

Maximal oxygen consumption (VO_2_max) was estimated through the 20-meter shuttle run test for aerobic fitness by Léger, et al,^20^ which shows reliability of r=0.89 for young individuals.^21^ During the test, the subjects must run on a flat surface, from one side to another (20 meters), with the pace being determined by a sound signal (beep). In this recording, by each sound signal, the test subject should have covered the estimated 20 meters. The signal frequency is gradually increased, as well as the running velocity, which must keep pace with the signal. The running speed increases 0.5km/h every 1 minute, starting with a speed of 8.5km/h.

The test is terminated when the test subject fails to achieve the 20 meters before the beep for two consecutive times, or gives up due to fatigue. The number of the last completed lap or the time in minutes must be recorded to estimate VO_2_max using the equation: *VO* *_2_* *max (ml.kg* *-* 1 *.min* *-* 1 *)=31.025+3.238X* *_1_* *-3.248X* *_2_* *+0.1536X* *_1_* *X* *_2_*, wherein X_1_ is the maximum speed achieved in the test (km/h), and X_2_ is the age in years. Male and female adolescents were classified as having adequate or inadequate levels of aerobic fitness according to the cutoff points suggested in FITNESSGRAM, which vary according to gender and age.^22^

Measurements of body weight and height were collected, according to standardized procedures,^23^ and body mass index (BMI) was calculated, classifying the individual according to the cutoff points of the International Obesity Task Force,^24^ which vary according to gender and age as normal weight, overweight, and obesity, with the latter two grouped and classified as excess weight. Only five adolescents in the sample were classified as having low weight and, due to low frequency, they were categorized as normal weight.

In the descriptive analysis of the variables, means, standard deviations, and frequency distribution were used. Normality of the data was verified using histograms of sample distribution; the variables age and lumbar strength testing did not show normal distribution. However, as the sample size is large, the theory described by the central limit theorem was used, which states that the frequency distribution of sample means tends to increasingly approach the normal distribution as the sample size increases. Thus, parametric tests were applied to the analysis of quantitative data through the Student's *t*-test for independent samples and one-way analysis of variance (ANOVA). Furthermore, to identify differences in the prevalence of low lumbar strength according to the independent variables, the chi-squared test of heterogeneity was applied. 

Binary logistic regression was used to assess associations between the outcome and sociodemographic indicators (gender, age, socioeconomic status), lifestyle (eating habits, physical activity, alcohol consumption), aerobic fitness, and presence or absence of excess weight, estimating the odds ratio (OR) and the 95% confidence interval. All variables were introduced in the adjusted model regardless of *p*-value in the crude analysis. The analysis was performed with a hierarchical method, following the recommendations of the literature,^25^ divided into three blocks: 1) sociodemographic variables (Distal); 2) lifestyle variables (Intermediate); and 3) Aerobic fitness and weight status (Proximal). Variables with *p-*value <0.20^26^ remained in the adjusted model when hierarchical adjusted analysis was performed. The level of significance was set at 5%.

Analyses were performed using Stata 11.0 software (Stata Corp. - College Station, Texas, USA), considering the design effect and sampling weight. The results were not stratified by gender because there was no interaction between gender and outcome.

## Results

A total of 5.8% of the adolescents were excluded from the study, as they were not in the pre-established age group. Thus, the sample consisted of 601 adolescents with a mean of 15.5 ± 1.1 years, with a predominance of female adolescents (55.2%, 95% CI: 51.2-59.2). Table 1 shows the mean values and standard deviation of age, anthropometric variables, BMI, aerobic fitness, and muscular endurance test in the lumbar strength testing of adolescents. Boys had higher values than girls of body weight, height, and aerobic fitness (*p*<0.05). There were no differences between genders regarding age, BMI, and muscle endurance at the lumbar strength testing.

**Table 1:**
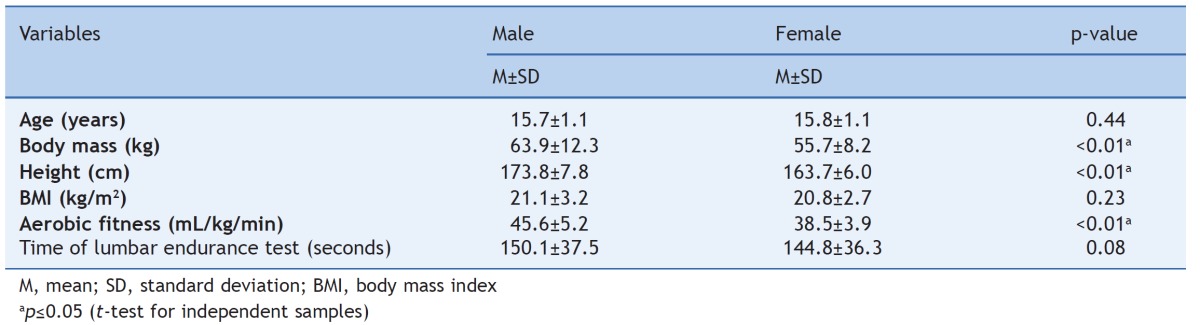
Mean and standard deviation of age, anthropometric variables, aerobic fitness, and lumbar endurance test time according to gender.

Table 2 shows that most of the sample was aged 16-17 years, of middle socioeconomic level. Of the students assessed, about two-thirds had an inadequate diet, and approximately half of the sample was insufficiently active, drank alcohol in excess, and had poor aerobic fitness. Additionally, one-in-seven students had excess weight. The adolescents who drank alcohol in excess, had adequate aerobic fitness levels, and had normal weight obtained better results at lumbar strength testing than the individuals who did not drink alcohol in excess, had low levels of aerobic fitness, and had excess weight, respectively (*p*<0.05).

**Table 2:**
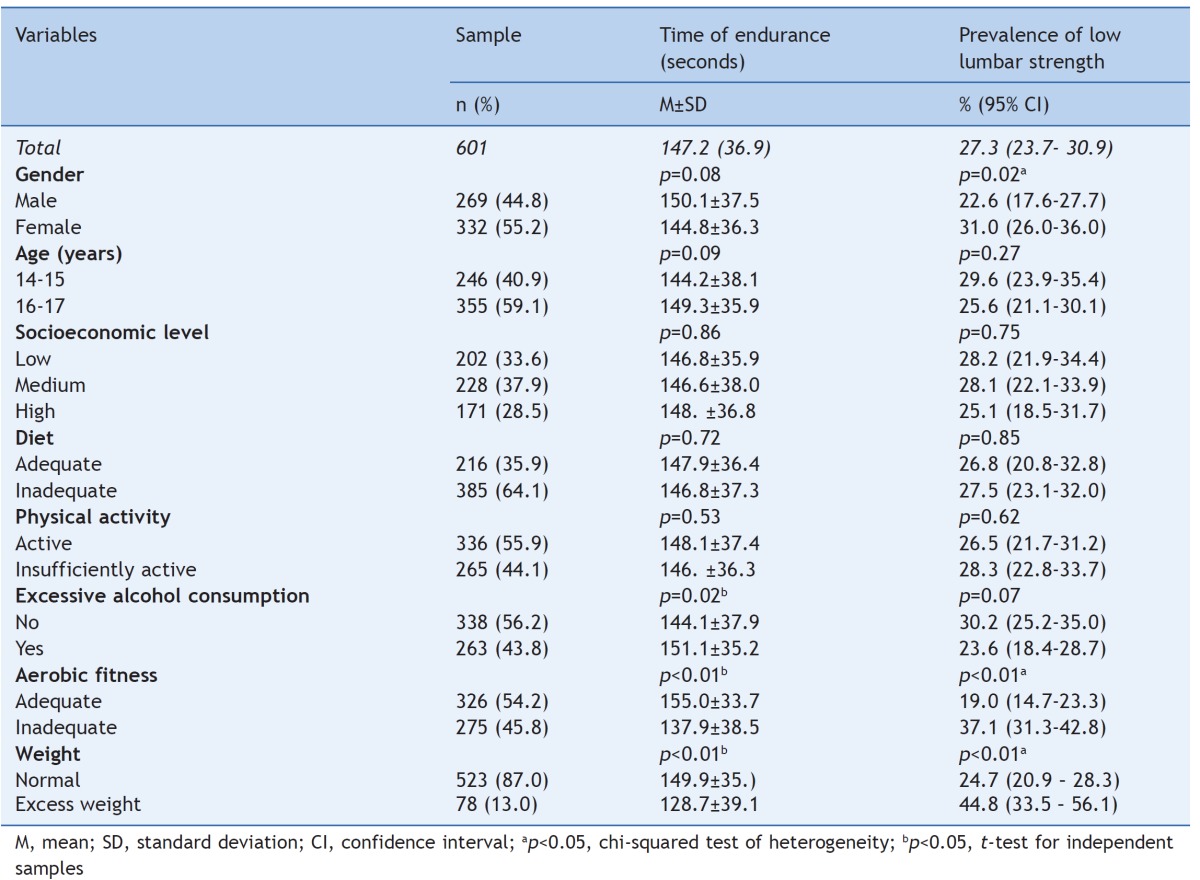
Sample distribution, time of lumbar endurance test, and prevalence of low levels of lumbar strength according to the independent variables among adolescents.

Both the crude and the adjusted analysis showed that the groups more likely to have low lumbar strength were females, adolescents with low levels of aerobic fitness, and those with excess weight (Table 3).

**Table 3:**
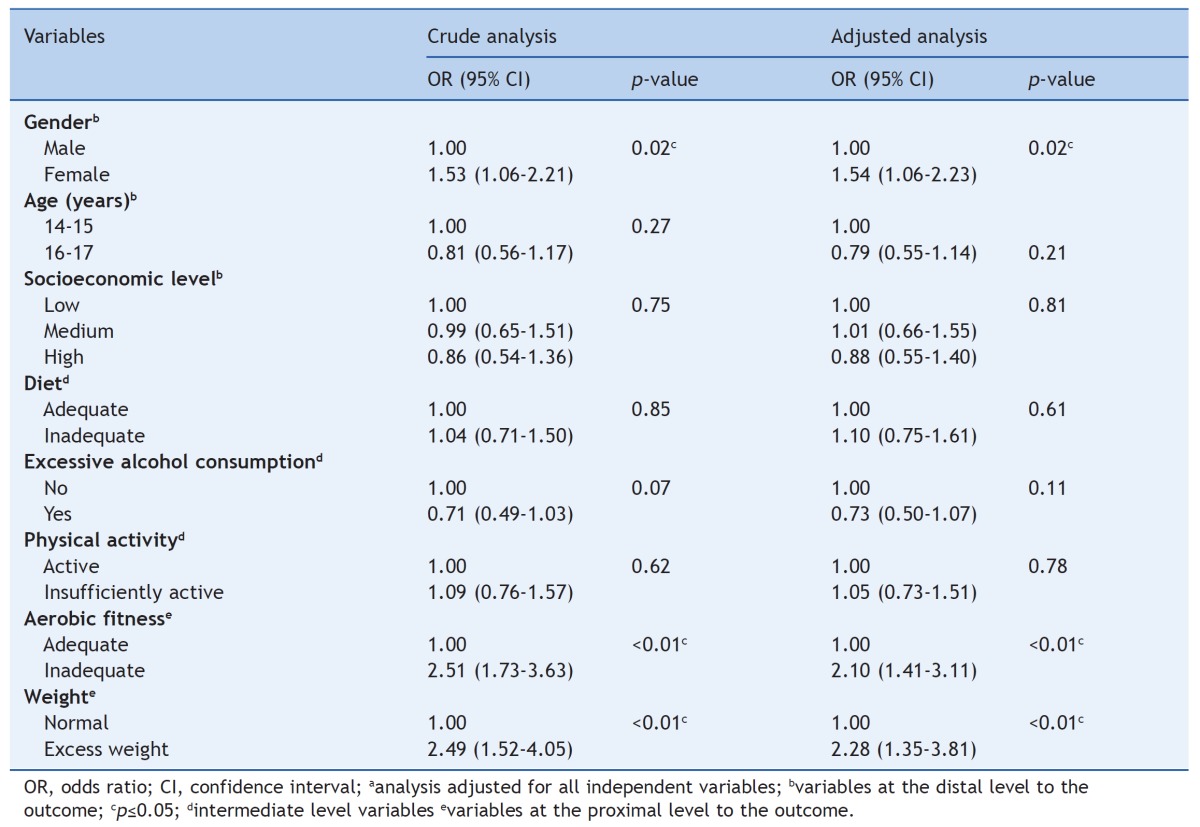
Odds ratios and 95% confidence intervals, crude and adjusted, between low level of lumbar strength and independent variables.

## Discussion

The aim of this cross-sectional study of a school-based population was to assess the prevalence and factors associated with low levels of lumbar strength in adolescents. The main findings of this research were that one-in-three teenagers had low levels of lumbar strength. The population subgroups most likely to have lower levels of lumbar strength were females, those with low aerobic fitness, and those with excess weight. This information is important to aid educational policies for school health by raising awareness of the importance of adequate posture and regular physical exercise throughout life.

The prevalence of low lumbar strength among the adolescents was 27.3%, higher in girls (31.0%) than in boys (22.6%). Lower levels of lumbar strength in female adolescents were also reported by other authors.^5^^,^ 7 A study carried out with 770 schoolchildren from Porto Alegre, Rio Grande do Sul, Brazil aimed to analyze factors associated with back pain in children and adolescents (aged 7-17 years) and found that female students complained more often of back pain than males, with this finding representing one of the possible factors responsible for back pain and lower levels of lumbar strength in girls.^5^

A school-based study carried out in Portugal with 924 adolescents aged 12 to 17 years reported lower levels of abdominal, lower back, and upper limb strength in female students when compared to males.^7^ One of the possible explanations for the difference between boys and girls may be related to regular physical activity during adolescence, which is typically practiced more often by boys, through playing games and competitive sports,^14^^,^ 27 which may develop higher levels of muscle strength and performance in boys. Furthermore, hormonal differences arising from the maturation process at the stage of adolescence favor a better performance of boys in physical tests, when compared to girls.^27^

Low levels of lumbar strength have been related in the literature as a risk for the emergence or development of some disorders, such as low back pain and possible postural distortions at different age ranges, increasing by six-fold the odds of possible lumbar complaints.^6^^,^ 7 Therefore, it is important for adolescents to receive guidance aiming to encourage regular physical activity to improve fitness, and muscular strength and endurance.

The present study found that adolescents with low levels of aerobic fitness were more likely to have low levels of lumbar strength. A study carried out in Portugal yielded similar results.^7^ One possible explanation for this finding may be the simultaneous presence of low levels of physical fitness in adolescents, that is, the young individuals with low levels of physical performance in a specific test are also more likely to have a similar result in another physical test, even if the other test assesses different components of physical fitness.^28^

In adolescence, the recommendations for physical activity indicate that adolescents should perform at least 60 minutes/day of moderate and vigorous activities. In general, these activities are of the aerobic type.^20^ However, good levels of aerobic fitness are also associated with adequate levels of muscular strength and endurance in adolescents. A possible explanation for this association is the concentration on sports practice, which constitutes the physical activity most often performed by adolescents. Such practice, while enhancing aerobic fitness, also helps to increase levels of muscular strength and endurance.^14^^,^ 27 ^,^ 28

The specific literature on lower back pain and lumbar strength recommends flexibility, muscular strength/endurance, and aerobic activities to reduce symptoms of lower back pain and treatment of this disease, as aerobic exercise also brings psychological benefits and decreases lumbar complaint symptoms.^29^

Regarding the variable obesity and its association with low lumbar strength, it was observed that the study results are close to the conclusions commonly found in the literature, i.e., that adolescents with excess weight have lower strength levels in the lumbar region.^3^^,^ 30 Further development of muscular strength/endurance is recognized as an important component of physical fitness in the prevention of chronic diseases, such as obesity. A longitudinal study conducted by the Aerobics Center Longitudinal Study with 1,506 men showed that participants with greater muscle strength had a lower risk of death.^30^

Excess weight significantly contributes to decreased muscle strength due to excess body fat and, consequently, to the presence of a smaller percentage of muscle mass, leading to the onset of symptoms associated with lumbar complaints.^3^^,^ 29 ^,^ 30 This accumulation of central fat promotes abdominal protrusion and thus, leads to a distension and weakness of the abdominal muscles. In individuals with excess weight, maintaining the balance and stability of the spine column during static posture and locomotion becomes more difficult because of the excess weight, the distribution of body mass, and the different anthropometric associations between anatomical structures of the trunk and limbs.^30^ Furthermore, excess body fat increases the risk of back strain and difficulties to recover from bouts of back pain because of the overall lack of physical fitness.^29^^,^ 30

The main study limitations include its cross-sectional design, which ultimately prevents the establishment of causal inference. It is noteworthy, however, that the sample size, representative of the public schools in the western Santa Catarina region, guaranteed the internal and external validity of the study. Therefore, the findings of this study allow for the conclusion that almost one-third of adolescents had low levels of lumbar strength. The female adolescents, individuals with inadequate levels of aerobic fitness, and those with excess weight had higher chances of having low lumbar strength. 
