# Supplementary material for: Factors associated with low levels of lumbar strength in adolescents in Southern Brazil
Source: Rev Paul Pediatr. 2014 Dec;32(4):360–6. doi: 10.1016/j.rpped.2014.05.001 (PMC4311790; doi:10.1016/j.rpped.2014.05.001)
Supplement: Translation

# Translation: Fatores associados aos baixos níveis de força lombar em adolescentes do Sul do Brasil☆

**Authors:** Diego Augusto Santos Silva, Eliane Cristina de Andrade Gonçalves, Leoberto Ricardo Grigollo, Edio Luiz Petroski

**Affiliations:** a Universidade Federal de Santa Catarina (UFSC), Florianópolis, SC, Brasil; b Fundação Educacional Unificada do Oeste de Santa Catarina (UNOESC), Chapecó, SC, Brasil

**Keywords:** Atividade motora, Aptidão física, Lombalgia, Saúde do adolescente, Coluna vertebral, Dor

## Abstract

**OBJETIVO:**

Verificar a prevalência e fatores associados a baixos níveis de força lombar em adolescentes.

**MÉTODO:**

Trata-se de um estudo transversal, com 601 adolescentes de 14 a 17 anos, estudantes de escolas públicas do oeste de Santa Catarina - Brasil. Analisou-se a força lombar por meio do teste de extensão lombar proposto pela Sociedade Canadense de Fisiologia do Exercício que propõe pontos de corte distintos para rapazes e moças. As variáveis independentes analisadas foram: sexo, idade, nível econômico, hábitos alimentares, consumo de álcool, atividade física e aptidão aeróbia. Para a análise dos dados utilizou-se a regressão logística uni e multivariável com nível de significância de 5%.

**RESULTADOS:**

A prevalência de baixos níveis de força lombar foi de 27,3%. Os subgrupos populacionais com maiores chances de baixos níveis de força lombar foram o sexo feminino (OR: 1,54; IC95%: 1,06-2,23), adolescentes com baixo nível de aptidão aeróbia (OR: 2,10; IC95%: 1,41-3,11) e com excesso de peso (OR: 2,28; IC95%: 1,35-3,81).

**CONCLUSÃO:**

Quase um terço dos estudantes de escolas públicas do Oeste Catarinense apresentaram baixos níveis de força lombar. Intervenções na população escolar devem ser adotadas com atenção especial aos estudantes do sexo feminino, aqueles com baixos níveis de aptidão aeróbia e com excesso de peso, pois foram os subgrupos populacionais com maiores chances de baixos níveis de força lombar.

## Introdução

A dor lombar, também conhecida como "lombalgia", é um dos desconfortos musculoesqueléticos mais comuns em adolescentes e adultos. No entanto, o diagnóstico nem sempre é específico, sendo considerada uma doença multifatorial.^1^

 Estudos apresentam uma grande variabilidade da estimativa da prevalência de queixas lombares entre a população jovem, variando de 1,1%^2^ a 66,2%.^3^ No Brasil, as pesquisas revelaram que a prevalência de adolescentes com dores nas costas variou de 19,5%^4^ a 31,6%.^5^

As principais causas de dores lombares nos escolares podem estar relacionadas à sobrecarga crescente como o suporte de mochilas escolares de maneira inadequada, permanência na posição sentada por períodos prolongados, prática de esporte de alta intensidade e baixos níveis de força lombar. A sobrecarga de mochilas escolares, o sedentarismo, a prática excessiva de esportes e os baixos níveis de força lombar, quando associados à obesidade e a hábitos posturais inadequados, maximizam a ocorrência de lombalgias.^6^^,^ 7

Um estudo de coorte realizado por Vital *et al* 8 avaliou 215 escolares portugueses e concluiu que baixos níveis de força muscular dos extensores/flexores do tronco estão associados à maior prevalência de dor lombar e a à baixa flexibilidade dos músculos posteriores da coxa. Resultados semelhantes foram encontrados em estudos longitudinais como o de Lee *et al*,^9^no Japão, com 67 jovens entre 17 e 19 anos e no de Sjolie & Ljunggren,^10 ^na Noruega, com 85 adolescentes com média de 14 anos de idade, que observaram associação de baixos níveis de força lombar com níveis elevados de IMC e histórico familiar de lombalgia. No Brasil, estudo transversal de Martins *et al*,^11^ com 60 adolescentes de 15 a 18 anos de idade, identificou que aqueles com hiperlordose lombar possuíam baixos níveis de força lombar. 

Os agravos acarretados pelos baixos níveis de força lombar acabam por interferir na qualidade de vida dos adolescentes e outros aspectos relacionados à saúde e em consideráveis gastos públicos para o tratamento e reabilitação desses danos.^7^^,^ 9 Porém, ainda não há estudos, na América Latina, com amostra representativa de adolescentes que analisassem simultaneamente a associação de baixos níveis de força lombar com fatores demográficos, socioeconômicos e de estilo de vida. Assim, tendo em vista que baixos níveis de força lombar se associam com o surgimento de dores lombares e que o aparecimento precoce de perturbações na coluna acomete parte dos indivíduos na vida adulta, torna-se relevante analisar os fatores associados aos baixos níveis de força lombar a fim de prevenir o surgimento de fatores de riscos pré-determinantes para as lombalgias. Para tanto, o objetivo deste estudo foi verificar a prevalência e fatores associados a baixos níveis de força lombar em adolescentes.

## Métodos

O estudo foi desenvolvido no Oeste Catarinense, que é composto por 13 municípios. A região oeste é uma das mesorregiões do estado brasileiro de Santa Catarina e possui índice de desenvolvimento humano (IDH) de 0.807.^12^. A principal cidade é Joaçaba, considerada o polo econômico e político do Meio Oeste catarinense, com população estimada de 25.322 habitantes.^13^

Esse estudo analítico transversal de base escolar teve como população alvo os escolares de 14 a 17 anos matriculados em escolas públicas estaduais e foi realizado no segundo semestre de 2008. O estudo foi aprovado pelo Comitê de Ética em Pesquisa com Seres Humanos da Universidade do Oeste de Santa Catarina (Parecer número 079/08).

Todas as 13 cidades da região foram incluídas no plano amostral, que foi determinado em dois estágios: 1) estratificado por escolas públicas de ensino médio (n=18) e 2) conglomerado de turmas. No estágio 1, consideraram-se somente as escolas com mais de 150 alunos matriculados (n=17). Além disso, nas cidades que possuíam mais de uma unidade de ensino, optou-se pela que tinha maior quantidade de alunos. Assim, a maior escola de cada município foi analisada. No estágio 2, foram convidados a participar do estudo todos os adolescentes do ensino médio que estavam presentes em sala de aula no dia da coleta de dados.

Para o cálculo amostral, adotou-se prevalência desconhecida para o desfecho (igual a 50%), erro tolerável de cinco pontos percentuais, nível de confiança de 95%, efeito de delineamento de 1,5, acrescentando 10% para possíveis perdas e recusas. Considerando que no Meio-Oeste Catarinense 4.582 escolares formavam o ensino médio, estimou-se uma amostra de 585 adolescentes. Porém, como foram convidados a participar da pesquisa todos os adolescentes pertencentes ao conglomerado de turmas, a amostra final foi composta por 635 adolescentes. 

Ao considerar os parâmetros de poder do estudo como de 80%, nível de confiança de 95% e número de sujeitos em cada categoria das variáveis independentes, o tamanho de amostra pôde detectar odds ratio acima de 1,4, e abaixo de 0.6 como fatores de risco e proteção, respectivamente, na análise bruta.

Definiu-se como elegível estar matriculado na rede estadual de ensino, encontrar-se na sala de aula no dia da coleta e ter de 14 a 17 anos de idade. Considerou-se recusa o adolescente não querer participar da pesquisa. Foi considerada perda amostral a falta de preenchimento no questionário de uma ou mais variáveis analisadas neste estudo ou a não realização de um ou mais testes físicos.

A variável dependente foi a força lombar. O teste empregado para avaliar os níveis de força na região lombar é um teste isométrico proposto pela Sociedade Canadense de Fisiologia do Exercício por meio da bateria descrita na *Canadian Physical Activity, Fitness and Lifestyle Approach (CPAFLA)*.^14^ O teste de extensão e sustentação do tronco, isometria dorsal, foi realizado sobre um banco no qual o avaliado deita-se em decúbito ventral, descansando sobre o banco apenas as pernas e o quadril, tendo as pernas apoiadas por uma corda preparada com acolchoamento nas regiões posterior da coxa e da perna. Ao sinal do avaliador, o avaliado eleva o tronco até a horizontalidade com as pernas, permanecendo pelo máximo de tempo queo avaliado for capaz nesta posição. O teste era encerrado quando o avaliado sedia o tronco ou sustenta-o até o tempo máximo de três minutos (180 segundos). Adolescentes do sexo masculino e feminino foram classificados com níveis adequados ou inadequados (baixos) de força lombar conforme os pontos de corte sugeridos no *CPAFLA, *que variam conforme o sexo e a idade.^14^

As informações sociodemográficas (sexo, idade e nível econômico) foram coletadas por meio de um questionário autoadministrado em sala de aula. A variável idade foi coletada de forma contínua e, posteriormente, categorizada em 14-15 anos e 16-17 anos. O nível econômico foi identificado pelo questionário da Associação Brasileira de Empresas de Pesquisa15 que divide a população brasileira em cinco classes econômicas, por ordem decrescente de poder de compra ("A1", "A2", "B1", "B2", "C1", "C2", "D" e "E"). No presente estudo, agruparam-se as categorias "A1", "A2" e "B1", considerado nível econômico alto; "B2", nível médio, e "C1", "C2" e "D" em nível baixo. Nenhum aluno foi classificado na classe "E". Optou-se por essa classificação devido à homogeneidade amostral nessas três categorias. 

Os hábitos alimentares dos escolares foram verificados a partir do item que integra o *Fantastic Life Style Questionnare*, traduzido e validado para o Brasil.^16^ O item tinha a seguinte sentença: Eu como uma dieta balanceada. Este item apresentava como opções de resposta: 1) quase nunca 2) raramente 3) algumas vezes 4) com relativa frequência e 5) quase sempre. Foi considerado como alimentação adequada quem respondeu as opções 4 e 5. O instrumento apresenta explicação do que vem a ser uma dieta balanceada com diferentes porções de grãos, cereais, frutas, vegetais, derivados do leite, carnes e semelhantes que variam conforme idade e sexo.

O comportamento em relação à atividade física foi analisado por meio do instrumento dos Estágios de Mudança de Comportamento (EMC) relacionados à atividade física,^17^ os quais classificam os sujeitos em um dos cinco estágios: (1) Pré-Contemplação (o sujeito não pretende modificar seu comportamento num futuro próximo); (2) Contemplação (existe a intenção de mudar, mas não imediatamente); (3) Preparação (sujeitos que não estão engajados em AF de forma regular, mas que pretendem se engajar nos próximos 30 dias); (4) Ação (ativos regularmente a menos de seis meses); (5) Manutenção (ativos de maneira regular a, no mínimo, seis meses). A partir da resposta do EMC, os alunos foram classificados em um estado de comportamento "insuficientemente ativo", estágios 1, 2 e 3, e "ativo", estágios 4 e 5. Evidências de eficácia, sensibilidade e especificidade desta classificação podem ser obtidas na literatura.^18^ Considerou-se como atividade física qualquer movimento corporal produzido pela musculatura esquelética e que resulta em gasto energético acima dos níveis de repouso.^19^ Considerou-se como atividade física regular as recomendações para adolescentes que afirmam que todos devem participar de atividades de intensidade no mínimo moderada por, no mínimo, 60 minutos pelo menos cinco dias na semana.^20^

O consumo excessivo de álcool caracterizado pelo *Binge Drinking, *que expressa o beber excessivo pelos jovens, foi investigado por um item do questionário *Fantastic Life Style*.^16^ O item é o seguinte: Eu bebo mais de 4 doses em uma ocasião. Este item apresentava como opções de resposta: 1) quase diariamente 2) com relativa frequência 3) Ocasionalmente 4) Quase nunca e 5) Nunca. Foram considerados como não consumidores excessivos de álcool sujeitos que responderam a opção 4 e 5, sendo os demais considerados consumidores excessivos.

O consumo máximo de oxigênio (VO_2_máx) foi estimado pelo teste de aptidão aeróbia de 20 metros de Léger *et al* 20 ^,^ que apresenta confiabilidade de r=0,89 para jovens.^21^ Durante o teste, os sujeitos devem correr em uma superfície plana, de um lado para outro (20 metros), com o ritmo determinado por gravação sonora. Nesta gravação, a cada sinal o avaliado deverá ter percorrido 20 metros. A frequência do sinal vai aumentando gradualmente, assim como a velocidade de corrida que deve acompanhar o ritmo do sinal. A velocidade de corrida aumenta 0,5 km.h^-^ 1 a cada 1 minuto, iniciando com velocidade de 8,5 km.h^-^ 1. O teste é encerrado quando, o sujeito não consegue atingir por duas vezes consecutivas os 20 metros antes do sinal sonoro ou quando desiste por fadiga. O número da última volta completada ou o tempo em minutos deve ser registrado para estimar o VO_2_máx por meio da equação: *VO* *_2_* *máx (ml.kg* *-* 1 *.min* *-* 1 *)=31.025+3.238X* *_1_* *-3.248X* *_2_* *+0.1536 X* *_1_* *X* *_2_*. Em que, X_1_ é a velocidade máxima obtida no teste (km.h^-^ 1), e X_2_ é a idade em anos. Adolescentes do sexo masculino e feminino foram classificados em níveis adequados ou inadequados de aptidão aeróbia conforme os pontos de corte sugeridos no *FITNESSGRAM *que variam conforme o sexo e a idade.^22^

Coletaram-se medidas de peso corporal e a estatura, conforme procedimentos padronizados,^23^ e calculou-se o índice de massa corporal (IMC), classificando o indivíduo conforme os pontos de corte da *International Obesity Task Force*,^24^ que variam de acordo com o sexo e a idade, em peso normal, sobrepeso e obesidade, sendo as duas últimas agrupadas e classificadas em excesso de peso. Somente cinco adolescentes da amostra foram classificados como baixo peso e, devido à baixa frequência, os mesmos foram categorizados como peso normal.

Na análise descritiva das variáveis foram utilizadas médias, desvios padrão e distribuição de frequências. Foi verificada a normalidade dos dados por meio de histogramas de distribuição amostral e as variávelisidade e teste de força lombar não apresentaram distribuição normal. Porém, como o tamanho de amostra é grande, fez-se uso da teoria descrita no Teorema do Limite Central que afirma que a distribuição de frequências das médias amostrais tende a se aproximar cada vez mais da distribuição normal à medida que o tamanho da amostra aumenta. Dessa forma, aplicaram-se os testes paramétricos nas análises dos dados quantitativos por meio do teste "t" de Student para amostras independentes e análise de variância (ANOVA) one way. Ademais, para identificar diferenças na prevalência de baixos níveis de força lombar de acordo com as variáveis independentes, aplicou-se o teste do qui-quadrado de heterogeneidade. 

Empregou-se a regressão logística binária para examinar as associações entre o desfecho com os indicadores sociodemográficos (sexo, idade, nível econômico), estilo de vida (hábitos alimentares, atividade física, consumo de álcool), aptidão aeróbia e presença ou não de excesso de peso, estimando-se a odds ratio (OR) e o intervalo de confiança de 95%. Todas as variáveis foram introduzidas no modelo ajustado independente do *p*-valor na análise bruta. A análise foi realizada de forma hierarquizada, seguindo as recomendações da literatura,^25^ sendo dividida em três blocos: 1) variáveis sociodemográficas (Distal); 2) variáveis do estilo de vida (Intermediário) e; 3) aptidão aeróbia e status do peso (Proximal). Permaneceram no modelo ajustado as variáveis com *p*-valor <0,20^26^ quando realizada a análise ajustada hierarquizada. O nível de significância foi estabelecido em 5%. 

As análises foram realizadas no software Stata 11.0 (STATA Corp. College Station, Texas USA), considerando o efeito de delineamento e o peso amostral. Os resultados não foram estratificados por sexo porque não houve interação entre o sexo e o desfecho. 

## Resultados

Foram excluídos da pesquisa 5,8% dos adolescentes por não estarem na faixa etária delimitada. Assim, a amostra foi composta por 601 adolescentes com média de 15,5±1,1 anos de idade, predominado os adolescentes do sexo feminino (55,2%; IC95%: 51,2-59,2). Na tabela 1 estão descritos os valores médios e o desvio padrão da idade, variáveis antropométricas, IMC, aptidão aeróbia e o teste de resistência muscular no teste de força lombar dos adolescentes. Os meninos apresentaram valores mais elevados do que as meninas para peso corporal, estatura e aptidão aeróbia (*p<*0,05). Não houve diferenças entre os sexos para idade, IMC e tempo de resistência muscular para o teste de força lombar.

**Tabela 1:** Valores de média e desvio padrão da idade, variáveis antropométricas, aptidão aeróbia e tempo de resistência no teste de força lombar de acordo com o sexo

Na tabela 2 observa-se que a maior parte da amostra tinha 16-17 anos e nível econômico médio. Dos estudantes investigados, por volta de dois terços apresentavam dieta inadequada, aproximadamente metade da amostra era insuficientemente ativa, consumia bebidas alcoólicas em excesso e tinha baixa aptidão aeróbia. Ademais, um a cada sete estudantes apresentava excesso de peso. Os adolescentes que consumiam bebidas alcoólicas em excesso, que tinham níveis adequados de aptidão aeróbia e eram eutróficos tiveram melhor desempenho de resistência muscular no teste de força lombar que os jovens que não consumiam bebidas alcoólicas em excesso, que tinham níveis baixos de aptidão aeróbia e aqueles com excesso de peso, respectivamente (*p<*0,05). 

**Tabela 2:** Distribuição da amostra, tempo de resistência muscular no teste de força lombar e prevalência de baixos níveis de força lombar de acordo com as variáveis independentes entre os adolescentes

Na análise bruta quanto na ajustada, identificoum-se que os grupos com maiores chances de baixos níveis de força lombar foram o sexo feminino, adolescentes com baixo nível de aptidão aeróbia e com excesso de peso (Tabela 3).

**Tabela 3:** Razões de chances e intervalos de confiança de 95%, bruto e ajustado, entre baixos níveis de força lombar e variáveis independentes

## Discussão

O objetivo desse estudo transversal de base escolar foi verificar a prevalência e fatores associados a baixos níveis de força lombar em adolescentes. Os principais achados desta pesquisa foram que um em cada três adolescentes apresentou baixos níveis de força lombar. Os subgrupos populacionais com maiores chances de mostrar baixos níveis de força lombar foi o sexo feminino e aqueles adolescentes que apresentavam baixa aptidão aeróbia e excesso de peso. Essas informações são importantes para subsidiar políticas educacionais de saúde do escolar por meio da conscientização da importância de uma postura adequada e da prática regular de exercícios físicos por toda a vida. 

A prevalência encontrada para o baixo nível de força lombar dos adolescentes foi de 27,3%, sendo maiores nas meninas (31,0%) do que nos meninos (22,6%). Menores níveis de força lombar em adolescentes do sexo feminino também foram relatados por outros autores.^5^^,^ 7 Pesquisa realizada com 770 escolares de Porto Alegre, Rio Grande do Sul, Brasil teve como objetivo analisar fatores associados à dor lombar em crianças e adolescentes (7 a 17 anos) e encontrou que estudantes do sexo feminino se queixavam mais de dores lombares que os do sexo masculino, sendo este achado um dos possíveis fatores responsaveis pelas dores lombares os menores niveis de força lombar nas moças.^5^ Investigação de base escolar desenvolvida em Portugal com 924 adolescentes de 12 a 17 anos relatou menores níveis de força abdominal, lombar e de membros superiores nos estudantes do sexo feminino, em comparação ao masculino.^7^ Umas das possíveis justificativas da diferença entre meninos e meninas pode estar relacionada à prática de atividade física regular na adolescência que, geralmente, é mais praticada por meninos, por meio de brincadeiras e jogos e esportes competitivos,^14^^,^ 27 o que pode desenvolver maiores níveis de força e desempenho muscular nos rapazes. Ademais, diferenças hormonais decorrentes do processo maturacional na fase da adolescencia favorecem um melhor desempenho em testes físicos pelos rapazes em comparação às moças.^27^

Os baixos índices de força lombar têm sido relacionados, na literatura, como risco para o surgimento ou desenvolvimento de algumas disfunções como lombalgia e possíveis distorções posturais em diferentes faixas etárias, elevando em seis vezes as chances de possíveis queixas lombares.^6^^,^ 7 Para tanto, é importante que os adolescentes recebam orientações com intuito de incentivar a prática regular de atividade física, a fim de melhorar o condicionamento físico e a força e resistência muscular.

O presente estudo encontrou que adolescentes com baixos níveis de aptidão aeróbia apresentaram maiores chances de terem baixos níveis de força lombar. Pesquisa desenvolvida em Portugal encontrou resultado semelhante.^7^ Uma possível justificativa para esse achado pode esta centrada na presença simultânea de baixos níveis de aptidao física em adolescentes, ou seja, aqueles jovens que apresentam baixos níveis de desempenho físico em um teste específico também têm maiores chances de apresentar o mesmo resultado em outro teste físico, mesmo que tais testes empregados avaliem diferentes componentes da aptidao física.^28^

Na adolescência, as recomendações para a prática de atividade física indicam que, diariamente, os adolescentes devem fazer no mínimo 60 minutos de atividades moderadas e vigorosas. Em geral essas atividades são do tipo aeróbias.^20^ Porém, bons níveis de aptidão aeróbia também estão associados a níveis adequados de força e resistência muscular em adolescentes. Possível justificativa para essa associação está centrada na prática de esportes, que se constituem na atividade física mais realizada pelos jovens. Tal prática, além de aprimorar a aptidão aeróbia, também auxilia nos níveis de força e resistência muscular.^14^^,^ 27 ^,^ 28 A literatura específica sobre lombalgia e força lombar recomenda atividades de flexibilidade, força/resistência muscular e atividades aeróbias para diminuir sintomas de dores lombares e tratamento dessa enfermidade, pois o exercício aeróbio traz benefícios psicológicos e diminuição dos sintomas de queixas lombares.^29^

Referente a variável obesidade e sua relação com baixos níveis de força lombar, foi constatado que o estudo se aproxima das conclusões comumente encontradas na literatura, ou seja, que os adolescentes com excesso de peso possuem menores níveis de força na região das costas.^3^^,^ 30 Um maior desenvolvimento da força/resistência muscular é reconhecido como importante componente da aptidão física na prevenção de doenças crônicas como a obesidade. Uma pesquisa longitudinal realizada pelo *Aerobics Center Longitudinal Study* com 1.506 homens revelou que os participantes com maior força muscular apresentaram menor risco de morte.^30^ O excesso de peso contribui significativamente na diminuição da força muscular devido ao excesso de gordura corporal e, consequentemente, à presenca de menores percentuais de massa muscular, levando ao aparecimento de sintomas associados a queixas lombares.^3^^,^ 29 ^,^ 30 Esse acúmulo de gordura central promove a protusão do abdome e, assim, leva a uma distensão e fraqueza da musculatura abdominal. Nos indivíduos com excesso de peso, a manutenção do equilíbrio e da estabilidade da coluna durante a postura estática e a locomoção se torna mais difícil em virtude do excesso de peso, da distribuição da massa corporal e das relações antropométricas diferenciadas entre as estruturas anatômicas do tronco e dos membros.^30^ Ademais, o excesso de gordura corporal aumenta os riscos de distensão nas costas e dificuldades para recuperar-se de crises de dores lombares por causa do estado geral da falta de condicionamento físico.^29^^,^ 30

Como principais limitações do estudo, destacam-se: o delineamento transversal, o que acaba por impedir o estabelecimento da inferência causal. Vale ressaltar, entretanto, que o tamanho da amostra, representativo das escolas públicas da região oeste catarinense, garantiu a validade interna e externa da pesquisa. Assim, Com os achados do presente estudo pode-se concluir que quase um terço dos adolescentes apresentaram baixos níveis de força lombar. Os indivíduos do sexo feminino, aqueles com níveis inadequados de aptidão aeróbia e os que apresentavam excesso de peso tiveram maiores chances de baixos níveis de força lombar.
